# Dementia Caregiver Experiences and Recommendations for Using the Behavioral and Environmental Sensing and Intervention System at Home: Usability and Acceptability Study

**DOI:** 10.2196/30353

**Published:** 2021-12-06

**Authors:** Martha Smith Anderson, Azziza Bankole, Nutta Homdee, Brook A Mitchell, Grace E Byfield, John Lach

**Affiliations:** 1 Department of Health Care Innovation and Implementation Science Virginia Tech Carilion School of Medicine Roanoke, VA United States; 2 Department of Psychiatry and Behavioral Medicine Virginia Tech Carilion School of Medicine Roanoke, VA United States; 3 Center for Research and Innovation, Faculty of Medical Technology Mahidol University Bangkok Thailand; 4 Virginia Tech Carilion School of Medicine Roanoke, VA United States; 5 Department of Genetics University of North Carolina at Chapel Hill Chapel Hill, NC United States; 6 Department of Electrical and Computer Engineering The George Washington University Washington, DC United States

**Keywords:** dementia, agitation, sensors, smart health, wearable technology, just-in-time notifications, caregiver, dyad, home-based, qualitative

## Abstract

**Background:**

Caregiver burden associated with dementia-related agitation is one of the most common reasons for a community-dwelling person living with dementia to transition to a care facility. The Behavioral and Environmental Sensing and Intervention (BESI) for the Dementia Caregiver Empowerment system uses sensing technology, smartwatches, tablets, and data analytics to detect and predict agitation in persons living with dementia and to provide just-in-time notifications and dyad-specific intervention recommendations to caregivers. The BESI system has shown that there is a valid relationship between dementia-related agitation and environmental factors and that caregivers prefer a home-based monitoring system.

**Objective:**

The aim of this study is to obtain input from caregivers of persons living with dementia on the value, usability, and acceptability of the BESI system in the home setting and obtain their insights and recommendations for the next stage of system development.

**Methods:**

A descriptive qualitative design with thematic analysis was used to analyze 10 semistructured interviews with caregivers. The interviews comprised 16 questions, with an 80% (128/160) response rate.

**Results:**

Postdeployment caregiver feedback about the BESI system and the overall experience were generally positive. Caregivers acknowledged the acceptability of the system by noting the ease of use and saw the system as a fit for them. Functionality issues such as timeliness in agitation notification and simplicity in the selection of agitation descriptors on the tablet interface were identified, and caregivers indicated a desire for more word options to describe agitation behaviors. Agitation intervention suggestions were well received by the caregivers, and the resulting decrease in the number and severity of agitation events helped confirm that the BESI system has good value and acceptability. Thematic analysis suggested several subjective experiences and yielded the themes of usefulness and helpfulness.

**Conclusions:**

This study determined preferences for assessing caregiver strain and burden, explored caregiver acceptance of the technology system (in-home sensors, actigraph or smart watch technology, and tablet devices), discerned caregiver insights on the burden and stress of caring for persons living with dementia experiencing agitation in dementia, and solicited caregiver input and recommendations for system changes. The themes of usefulness and helpfulness support the use of caregiver knowledge and experience to inform further development of the technology.

## Introduction

### Background

Caregiver burden associated with dementia-related agitation is one of the most common reasons for a community-dwelling person living with dementia to transition to a care facility. Agitation is a highly prevalent behavior and is one of the most persistent neuropsychiatric symptoms associated with dementia [1,2]. Several studies have examined the use of technology for the detection and prediction of agitation in dementia [3]. A review of smart health technologies for persons living with dementia and their caregivers found that most technologies address activities of daily living but not behavioral changes and fail to involve the end user’s experience in the development of products [3]. A review of home-based monitoring systems for early agitation detection that promotes the use of behavioral interventions calls to attention the fact that research must involve caregivers and persons living with dementia and be flexible enough to meet the need for individualization of the systems [4]. Management of disruptive behaviors for caregivers of persons living with dementia was identified in 8 of 118 mobile apps. In a discussion with 4 caregivers, only 2 apps were preferred as helpful, specific intervention strategies [5].

A design framework to guide smart health technology development in caregivers of persons living with dementia was based on a review of factors influencing the adoption of technology, including ethical issues, and identified challenges for both cognitive and physical decline [3]. Challenges of mobile app users in protecting privacy were identified within the theme of helplessness related to the overwhelming nature of the digital world [6]. In-home monitoring of persons living with dementia was studied for unobtrusive preferences in both formal and informal caregivers. Both felt potential benefits in more proactive responses to the needs of persons living with dementia [7]. The COVID-19 pandemic brought increased urgency in the development of remote monitoring systems, which are applicable to our focus on agitation with dementia.

### The Behavioral and Environmental Sensing and Intervention System

**Figure 1:**
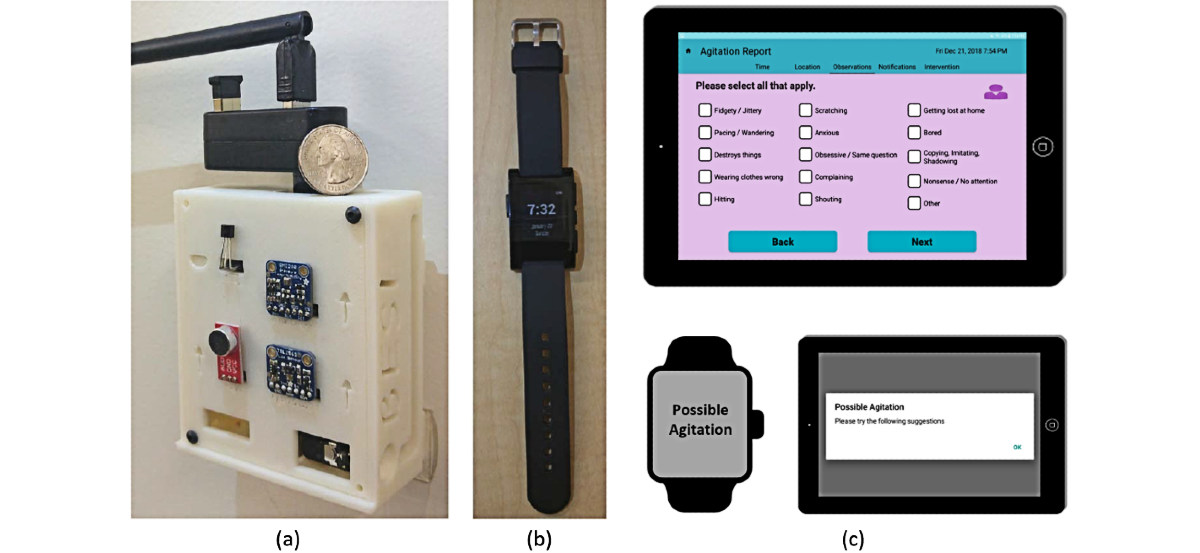
The Behavioral and Environmental Sensing and Intervention (BESI) system. The components of the system are (a) BESI environmental sensing nodes deployed in houses with people living with dementia, (b) a wearable sensor used to monitor behaviors of people living with dementia, and (c) tablet and wearable applications for caregiver interfacing, such as delivering agitation intervention suggestions.

The Behavioral and Environmental Sensing and Intervention (BESI) for Dementia Caregiver Empowerment system (Figure 1) for persons living with dementia and caregivers living together (dyads) at home uses sensing technology, smartwatches, tablets, and data analytics to detect and predict agitation in persons living with dementia [8]. The BESI project was a 3-phase study completed over 6 years with the goal of understanding the environmental and interpersonal factors that influence persons living with dementia agitation, caregiver stress, and the impact of agitation on the caregiver. The unit of study in the BESI project was the dyad. The BESI system was deployed in the dyads’ homes for 30- or 60-day trials. The 60-day trials included just-in-time notifications and dyad-specific intervention recommendations to caregivers that were based on the clinical assessment completed at intake, interviews with the dyad, and demographic information. This innovative system was designed to provide caregivers with a potential early warning for episodes of agitation and provided an opportunity for caregiver awareness and intervention before behavioral distress occurred, thus reducing caregiver burden and improving quality of life.

We conducted predeployment interviews with each dyad to assess the history of agitation, other neuropsychiatric symptoms, cognition, sleep, burden, self-efficacy, quality of life, depression, dementia staging, and functional assessment using standardized assessment tools. Postdeployment interviews were conducted on system value, usability, and acceptability. The BESI system showed that there is a valid relationship between dementia-related agitation and environmental factors and that caregivers prefer a home-based monitoring system [9].

In our follow-up study, implementing a Caregiver-Personalized Automated Non-Pharmacological Intervention System (CANIS) for dementia-associated agitation, we sought additional input from caregivers for a narrative of their experiences months after the completion of the BESI study. The interviews included open-ended response questions at the end of each category. The primary aim was to use information from caregiver interviews to inform technological preferences and assess insights and impact on caregiver mood and burden.

### Context and Objectives of the Study

Following the completion of the BESI study, we embarked on the CANIS study to help further the process of automating the personalized interventions developed with BESI. As part of BESI, phase 3, we evaluated several dementia-related interventions and suggestions and categorized them as intellectual stimulation or interpersonal communication. The intervention categories were accompanied by appropriate suggestions for interventions. For example, dementia-related suggestions in the interpersonal communications category could be *ask yes or no questions if possible* or *offer distracting activity during personal hygiene care, for example, hand them a washcloth to clean their face*. Our team linked the intervention categories to the individual responses of the clinical assessment tools in CANIS to create interventions and suggestions for each dyad that were customized to their needs.

The aim of this study is to determine preferences for assessing caregiver strain and burden, explore caregiver acceptance of the technology system (in-home sensors, actigraph or smart watch technology, and tablet devices), discern caregiver insights on the burden and stress of caring for persons living with dementia experiencing agitation in dementia, and study caregiver input and recommendations for system changes.

## Methods

### Recruitment

The study was conducted in southeastern United States. We mailed letters to caregivers who participated in BESI phases 2 and 3 to determine their interest in participating in CANIS. Only 4 responded to the letter, with 2 declining to participate and 2 who became our first interviewees. Our institutional review board subsequently granted permission to contact previous caregivers in their preferred method of communication (letters, phone calls, SMS text messages, or emails), as established in the BESI study. We obtained 10 agreements for interviews with these previous participants in the BESI study. Of these 10, 4 of the responding dyads had participated in phase 2 only, 4 in phase 3 only, and 2 dyads had participated in both the phases. Thus, 6 of the 10 original dyads from phase 2 and all 6 dyads from phase 3 participated in CANIS.

### Procedure

Written informed consent was obtained from all the participants. All 10 semistructured interviews, which were recorded, were conducted by 1 researcher (MSA). The research coordinator transcribed all 10 interviews. Of the 10 dyad participants, interviews for 7 were completed in person and for 3 were conducted over the phone. The interviews lasted approximately 75-100 minutes. These CANIS participants were invited to receive feedback from the initial BESI phase 2 caregivers, and 9 of the 10 caregivers agreed to this. The caregiver who declined chose to participate in the other semistructured interview questions that asked for thoughts and feelings in interactions with the BESI system. Participants were offered an honorarium of US $50. The study was approved by the health system’s Institutional Review Board (CANIS: IRB-19-517#).

### Data Analysis

Analysis began with the first author (MSA) reading and reviewing personal interview notes and transcriptions, repeating the review of each interview multiple times to determine the general nature of the material. Next, words and phrases related to similar ideas or constructs were identified. These findings were then coded, grouped into categories, and related to the group of interviews. Feedback topics were categorized, and the second author (AB) reviewed the material with back-and-forth discussions of categories and codes leading to condensation, subcategories, and subthemes. This process continued until the 2 authors agreed upon the findings [10-12] and determined the themes.

The Progressively Lowered Stress Threshold model [13,14] was applied conceptually to understand the impact of agitation in dementia. The Progressively Lowered Stress Threshold model demonstrates that increasing stress over time leads to outbursts and that changing the environment can help reduce stress and behavioral outbursts. Thus, recommendations for dementia agitation interventions need to be individualized to the dyad to be the most effective [13-15]. Gaining information about BESI system usability was essential in order to offer individualized recommendations.

Efforts to engage caregivers in the development of the system were made throughout the BESI study. One of the initial steps in the BESI study was to engage with caregivers in defining descriptive words for agitation and involved research team members attending multiple Alzheimer association support group meetings [15]. An original list of agitation descriptors from the Cohen-Mansfield Agitation Index [16,17] was shown to support group members in eliciting other word suggestions. The goal was to allow each dyad to capture its own unique descriptors of its experiences of agitation. In the early phases 1 and 2 of BESI, participant-oriented design decisions included the preferred method of communicating (email, SMS text message, or phone), more concise assessments of both members of the dyad, and determining of the layout of the home before the placement of sensors [8]. This study focused on caregiver input months after the initial participation. Although persons living with dementia were not interviewed here, they were involved throughout intake, assessment, and postdeployment in the BESI study. Most caregivers chose not to have the persons living with dementia present during the interviews. The research team has worked together for over 6 years and has consistently involved end users in developing and responding to changes in the technology. The importance of these follow-up interviews for thematic analysis is essential for guiding further understanding.

### Assessment Scales

An extended battery of standardized assessment scales was administered during the first home visit in the BESI study [8]. Geriatric clinicians, both a geriatric psychiatrist and 2 advanced practice nurses, administered all the tests. Assessments were selected for both the caregivers and the persons living with dementia. The goal of reducing burden or depression accompanied the technology goal of identifying early agitation to reduce problematic behavior escalation. In Multimedia Appendix 1, the assessment scales are listed by those measuring caregiver status and those measuring the status of the person living with dementia. Depression, sleep, confidence in caregiving, burden and strain, and caregiver distress were assessed as being related to caregivers [18-20,22,23]. Cognition and functional levels, neuropsychiatric symptomatology, agitation, depression, quality of life, and sleep quality were specific to persons living with dementia [17,21,23-29].

Automated intervention suggestions were piloted in the last 5 dyads in phase 3. The clinical team identified unique intervention recommendations based on the completed assessment scales and intake interviews. The suggested interventions were sent in response to system detection of an agitation event, even if the caregiver did not confirm the agitation. Notifications were sent to the caregivers on their smartwatches in all phase 3 deployments during the second 30 days. For deployment 1, the notification text asked, “Is this agitation,” and the caregiver’s response was “yes” or “no.” In the remaining deployments (ie, 2-6), the notification merely stated *Upcoming Agitation*.

### Qualitative Analysis

Interview transcriptions and personal interview notes were read and reviewed multiple times to determine the general nature and themes of the responses. *Meaning units* from prior BESI caregiver interviews guided the process. Meaning units were defined as categories of subjective opinions about participating in the research and asked for agreement or disagreement with those opinions. We then asked the participants for additional thoughts, allowing open-ended responses. Words and phrases were classified based on their relationships with similar ideas. Inductive analysis [10] led to the condensation of the transcripts into positive or negative responses. These findings were grouped into categories and related back to caregiver interviews. Researchers used an inductive approach in these interviews to seek insight and reflection on the initial themes. As the data were analyzed, themes consistent with caregivers’ subjective experiences were clarified. The authors reviewed the material and discussed the findings until they were in agreement. The interviews in this study sought reflections on the cascade of responses regarding caregivers’ subjective experiences, which are categorized in Textbox 1.

Positive and negative responses were reviewed to interpret the data. Whether the response was positive or negative, discussions between the first 2 authors determined whether the agreement was related to the interview question. Quotations from participants were studied and discussed back and forth to determine their thematic relevance.

Example quotes and meaning units of the subjective experiences of BESI phase 2 caregivers were shown to the phase 3 caregiver group. They were then asked, “Do you agree with this finding?” (with *yes* indicating a positive response) and “Do you have any additional thoughts on this?”

Understanding how caregivers felt about participating in the research was important in evaluating their acceptance of the technology and its presence in their homes. First, caregivers were asked to think back to their time of participation and recall whether they had negative or positive experiences. In total, 9 caregivers responded with positive comments, such as, “it made me stop and think; I try to view each day as a learning opportunity” and “anything that keeps everything quiet, calm or happy.”

Of these, 3 other positive responses mentioned the support of the team, and 2 addressed the future—*someone will benefit*. The 1 negative comment noted, “It was complicated to keep up with it all.” A range of experiences from *no difficulty* to *no help* was reported.

**Categorization of the caregivers’ subjective experiences.**

**Categorization of the caregivers’ subjective experiences**

1. No difficulty: covers a variety of brief responses regarding the use of Behavioral and Environmental Sensing and Intervention (BESI) technology or their impact on the behavior of persons living with dementia. This includes using the tablet, BESI application, in-home sensors, and smartwatches.
2. Functionality: describes the targeted actions required to record data in the BESI application or smartwatches; ease of use.
3. Recommendations: specific information about how the BESI system as a whole could be improved to make caregiving tasks easier or better customized to their needs.
4. Future capability: includes input on ways to maximize the usefulness of the BESI technology as a future product for caregivers of persons living with dementia.
5. Esthetics: most often, references to having in-home sensors mounted on their walls, including having sensors falling from walls and even damaging paint and wallpaper.
6. Intrusiveness: describes unusual behaviors in persons living with dementia related to having sensors in their homes.
7. No help: describes caregiver feelings about the BESI system being of no use in reducing the intensity or improving the challenging nature of caregiving.

In this study, we shared qualitative quotes from our earlier work [31], seeking input and asking caregivers if they agreed or disagreed with the statements. Interviewers then offered open-ended questions to gain additional thoughts and insights. Table 1 presents an example of coding in the thematic analysis of caregiver responses to the subjective experience.

**Table 1:** Caregiver-Personalized Automated Non-Pharmacological Intervention System (CANIS) example of coding in the thematic analysis of caregiver responses to the subjective experience.

Feedback topic	Phase 2 BESI^a^ qualitative quotes	Meaning units	Caregiver follow-up interviews: “Do you agree with this statement?”		Condensation	Subtheme	Theme
			Yes	No			
Incorporation and impact of all the aspects of the BESI technology on the behavior of persons living with dementia	“They were not a bother to use.”	No difficulty	8	1	Positive; because of functionality	Agreeability; ease of use	Usefulness
Caregiver perceptions of actions necessary to record data with the BESI technology	“If we were out, I sometimes did not remember exact time of agitation occurrence...At times I would forget to make a(n) entry upon return.”	Functionality	7	2	Negative; because of difficulty with functionality and burdensome	Burden; frustration; and negative ease of use	Usefulness
Customization of the BESI system to better serve caregiver needs	“Not much, maybe to be able to add to the choice on the Daily report page.”	Recommendations	8	2	Positive; because of functionality, ease of use, recommendations, and give “own thoughts”	Personalization; future potential	Helpfulness
Ways to maximize usefulness of the BESI technology as a future product for caregivers of persons living with dementia	“Ability to measure over time their attitude and activities that set off the agitation.”	Future capability	9	0	Positive; because of recommendations on functionality	Personalization; future potential	Helpfulness

^a^BESI: Behavioral and Environmental Sensing and Intervention.

## Results

### Demographics

Descriptive statistics and demographic information for the 10 unique caregivers interviewed and information about the person living with dementia in each dyad are presented in Table 2. Caregivers were mostly female (n=8) and were well-educated, 6 had bachelor’s degrees, and the other 4 had high school diplomas. The mean age was 65.80 years (SD 15.1 years; *R*=45). All caregivers were White despite efforts to recruit a diverse population. Days between deployment and the time of the CANIS interview indicate the most recent data collection in an earlier phase of the BESI study and ranged from 253 to 1076 days.

**Table 2:** Demographics and description of Caregiver-Personalized Automated Non-Pharmacological Intervention System (CANIS) dyads.

Caregiver characteristic			Deployment phase and dyad ID																							
			Phase 2 (P2)													Phase 3 predeployment (P3 pre)										
			D1^a^		D3^a^		D4		D6		D9		D10		D1^a^			D2		D3^a^		D4		D5		D6
Age (years)			73		78		82		56		70		43		79			77		74		60		80		37
Sex			Male		Female		Female		Female		Female		Female		Female			Male		Male		Female		Female		Female
Education level			BS^b^		BS		BS		BS		BS		HS^c^		BS			HS		BS		BS		HS		HS
Days between deployment and interview			—^d^		—		1029		1036		806		780		1076			456		1164		356		253		313
**Assessments**																										
	NPI-Q^e^ Symptom Severity	10		18		9		3		20		18		23			13		10		23		10		26	
	NPI-Q Caregiver Distress	11		27		14		3		24		24		31			11		15		23		48		37	
	CMAI-C^f^ frequency	35		69		45		60		92		69		119			54		46.5		86		50		100	
	CMAI-C behaviors	2		10		8		13		18		15		13			11		6		16		7		17	
	Cornell Scale for Depression in Dementia	5		15		6		4		15		21		23			20.5		7		12		11		15	
	Center for Epidemiologic Studies Depression Scale	4		26		13		19		33		10		18			4		6		14		13		40	
	Quality of Life-Alzheimer Disease	37		26		27		36		33		25		27			30		39		29		28		18	
	Pittsburg Sleep Quality Index	2		3		4		6		8		7		13			1		7		4		5		16.5	
	RSSE^g^-respite	54		50		66		98		24		30		20			56		83		100		40		0	
	RSSE-behavior	100		50		52.5		60		82		90		63			84		100		50		70		68	
	RSSE-thoughts	90		92		72.5		95		28		81.6		91			76		93		87.5		55		56.3	
	Zarit	21		18		20		16		35		14		13			9		23		15		24		34	
	Barthel	85		95		75		85		90		60		75			75		75		40		70		75	
	Caregiver Strain Index-CANIS only	—		—		5		6		9		10		7			8		8		12		8		12	

^a^Identical dyads.

^b^BS: bachelor’s degree.

^c^HS: high school.

^d^Dyad participated in both phase 2 and phase 3, thus more recent measures were used.

^e^NPI-Q: Neuropsychiatric Inventory Questionnaire.

^f^CMAI-C: Cohen Mansfield Agitation Inventory-Community form.

^g^RSSE: Revised Scale for Caregiving Self-Efficacy.

### Insights Into Caregivers per Assessment Scales

Caregiver assessments by the clinical team offered other insights into the aspects of the dyad. Caregiver burden and strain caused by agitation in the person living with dementia significantly contributed to transitions to care facilities. In the CANIS study, 2 persons living with dementia had moved to assisted living facilities since their participation in the BESI study. Both caregivers remained active in visiting and supporting the person living with dementia. In total, 1 person living with dementia died. The extended time from the BESI study to follow-up interviews in the CANIS study may have contributed to the evolution of placement or decline.

Caregivers reported agitation in the person living with dementia as a criterion for study participation and also mentioned it in the measures of the Cohen-Mansfield Agitation Inventory [23] and Neuropsychiatric Index [19]. All persons living with dementia scored positive for dementia with the Modified Mini Mental State Examination measure of cognitive function (mean 48.9, SD 28.42; *R*=83.00), where a score <79 indicates cognitive impairment. Functional Assessment Staging is another measure of cognition with scores ranging from normal to Alzheimer disease; all persons living with dementia scored ≥4, indicating mild dementia, (mean 6.7, SD 2.98; *R*=8.00).

Caregiver scores on the Zarit Burden Scale [20] showed a mean of 19.43 (SD 8.05; *R*=26; maximum score=48), with higher scores indicating greater burden. Of them, 1 caregiver rated burden as rare, 2 rated it as moderate, and 9 rated it as mild to moderate. None of the participants rated the burden as severe. Burden was not excessive in these caregivers.

The Caregiver Strain Index (CSI) [21] has recently been used in research on older adults. This scale was introduced in the CANIS study. We asked caregivers for their preference or if they found one more helpful or appropriate, as burden and strain are often major concerns in caregiving. In a large national study of older adults in caregiving roles [31], caregivers of persons living with dementia in the last 12 months of life had double the amount of strain, as measured by 2 national surveys. There was no clear preference for assessment in these caregivers, as the Burden (Zarit) scale was preferred by 5 caregivers and the CSI scale was preferred by 4 caregivers. For the CSI, a score of 7 or higher indicates a high level of caregiver strain (mean 8.5, SD 4.95; *R*=7). Caregivers rated a score of >7 7 times, indicating a greater burden than was measured with the Zarit scale, despite no clear stated preference between the scales. The CSI asks for responses as yes (score=2), sometimes (score=1), or no (score=0) and is summed. The Zarit is also summed but offers more choices as to whether burden occurs never (score=0), rarely (score=1), sometimes (score=2), quite frequently (score=3), or nearly always (score=4). A caregiver explained that there was no preference stating the following:

The first one (CSI) had questions that made me reflect that I am perhaps guilty about the issue. Although I can still answer it.

Others preferred the Zarit because it offered *more flexibility* with the larger range of responses or because it was *deeper and more relevant*. It is essential that burden and strain are addressed in dementia caregivers, whichever valid tool is selected.

Depression in caregivers was assessed using the Center for Epidemiological Studies Depression Scale [24]. Higher scores indicate more symptoms. The maximum level of depressive symptoms is 60. The mean depression score was 14.1 (SD 9.85; *R*=36); only 3 scored >25 to 40. The caregivers did not indicate that they were significantly depressed.

The Cornell Depression Scale [25] rates depression in persons living with dementia by caregiver observation of severity; ratings are unable to evaluate, absent (score=0), mild to intermittent (score=1), and severe (score=2). Scores >12 indicate probable depression in the person living with dementia. In total, 7 persons living with dementia scored >12. These persons living with dementia severity scored X=13.93, SD 6.28, and *R*=19. Caregivers perceived the person living with dementia to be depressed.

The presence of a burden and even mild depression in caregivers could impact their responses. Overall, the assessment scores did not reveal excess burden or depression in caregivers, even though they felt differently about the persons living with dementia. We assessed the quality of life of the persons living with dementia using the Quality of Life-Alzheimer Disease scale [26]. This instrument is based on caregiver input (X=29.10, SD 5.21; *R*=21). The scores indicate good ratings for the group.

Caregiver measures for confidence in 3 aspects of caregiving were measured using the Revised Scale for Caregiving Self-Efficacy [19]. Higher scores indicate greater confidence. The mean for the Revised Scale for Caregiving Self-Efficacy for obtaining respite was 51.70 (SD 34.53; *R*=100), for responding to disruptive behaviors was 71.95 (SD 16.54; *R*=50.00), and for controlling upsetting thoughts about caregiving was 73.59 (SD 21.42; *R*=67.00). Caregivers demonstrated high confidence, especially in the latter 2 categories. Obtaining respite was a midpoint mean and was supported by 8 caregivers spending up to 24 hours/day with the person living with dementia and the remaining 2 spending more than 12 hours/day. The opportunity to obtain respite is challenging in these time commitments of caregiving. Sleep, as measured by the Pittsburg Sleep Quality Index [21], was X=7.15, SD 4.55, and *R*=15.50, where scores >5 indicate poor sleep quality. Half of the caregivers rated the sleep quality of the person living with dementia as >5. Sleep difficulties and decreased confidence in obtaining respite may demonstrate the burden of caregiving. Impaired sleep quality is a known cause of additional stress for caregivers of persons living with dementia [2].

Caregivers expressed strain and burden, saying, “It’s just like nothing helps” and expounding on the particular difficulty with agitation and participating in the study, “That’s part of the burden. You have a to deal with the agitation, then recording it.” It is difficult to see changes in persons being cared for while losing social connections and privacy and often experiencing financial and physical changes [20,21], especially with functional changes in persons living with dementia at the end of life [22] or with disease progression.

Agitation in the person living with dementia, as measured by caregivers with the Cohen-Mansfield Agitation Inventory for frequency (X=72.15, SD 25.67; *R*=74.00; maximum 203) and behavior occurrence (X=12.40, SD 4.27; *R*=12.00; maximum 29), was in lower quantities. Neuropsychiatric symptom severity (X=15.64, SD 7.56; *R*=23.00; maximum 36) was mild to moderate, and distress experienced by the caregiver because of the symptoms (X=23.00, SD 13.23; *R*=45.00; maximum 60) was mild. Agitation can create a burden even if symptoms are not severe or very frequent [1], and it is one of the most significant symptoms of dementia leading to institutionalization [1].

### Themes

Analysis of caregiver interviews in the CANIS study regarding participation in research with the BESI system revealed themes of usefulness and helpfulness with subthemes of agreeability and ease of use. The theme of usefulness was derived from both positive feelings about the ease of use of the BESI system, including receiving automated intervention suggestions and negative feelings about the difficulties with functionality of the developing system, as indicated by burden and frustration. Caregiving burden and stress were demonstrated in caregivers giving lengthy feedback, deemed helpful by researchers, in the potential for the system but feeling that their input was negative.

The interviews comprised 16 questions. With the 10 caregivers, out of a possible 160 responses, there were 128 (80%) responses, which were transcribed. Overall, participating for 30-60 days in the BESI study was perceived positively. Caregivers expressed the desire to “give more information” and were future-oriented; “People are getting an idea of what I am going through.” Positively coded feedback supported the subthemes of agreeability and ease of use with caregivers’ sense of commitment to the technology.

The theme of helpfulness related to feedback on the potential of the system and was demonstrated by commitment to completing the surveys and allowing multiple interviews many months after the initial intake visit. Numerous detailed future recommendations from caregivers revealed their helpfulness in participating in the next phase of the system’s development and in their belief that the system would be beneficial. The future potential to detect agitation and give a *heads up* before it became a behavior difficulty spoke of the helpfulness that caregivers ascribed to the system.

Agreeability with receiving agitation notifications on the smartwatch and with consistent support for the research team was demonstrated. Future potential of the developing system was emphasized, and it supports users’ acceptance of the technology. Ease of use in the overall acceptability of and confidence in handling the technology was evident:

They [sensors] were not a bother to me.

I was actually called to fix things myself.

The themes of usefulness and helpfulness were supported by caregiver feedback, participation, and future recommendations.

### Caregiver Acceptance of the BESI Technology System

Caregiver responses to previous caregiver quotes were largely positive. Positive responses applied to sensors “not being a bother” in the home, recommendations of being able to make changes such as “to put my own thoughts in,” and to future capability, with all caregivers agreeing that the system would be of benefit. Ease of use was evident both in the negative—“It was a nuisance to run over to the table...every time I thought something was worthy”—and the positive—“it was good”—feedback regarding receiving notifications on the smartwatch about possible agitation.

Agreeability was evident with responders demonstrating acceptance of receiving the notifications indicating possible agitation. When asked, “When we sent notifications on your smartwatch asking about agitation, how did it affect you?”—70% (7/10) of the respondents replied that it was “no problem” or “it didn’t.” In contrast, 1 response was negative—“after a while, it was irritating since it was too late.” As to whether it increased their awareness of the situation, “yes” and “no” were answered equally, with 1 caregiver adding that it confirmed the agitation. Later in the study, automated suggestions or possible interventions were delivered at the end of each week. Moreover, 4 phase 2 participants did not receive any notifications, and thus did not respond to this question. Another 2 caregivers from phase 3 said they “hardly remembered them.” In total, 3 caregivers felt they were “no help” and only 1 found them helpful. This first attempt to deliver individualized interventions did not have a significant impact and was not useful or helpful at the time. Later, in these interviews, 70% (7/10) of respondents indicated positive feelings about the automated intervention suggestions with comments including:

It just made me more aware of what was going on and made me think: Do I need to do anything?

It was good,...would be helpful.

Something that could say “heads up, do something” would be nice.

The usefulness and helpfulness of the technology were also affirmed in interviews with 90% (9/10) of the caregivers agreeing on the future capability of the BESI system. Most caregivers did not notice a change in agitation frequency over the course of deployment. Moreover, 1 caregiver offered additional thoughts about the unique characteristics of agitation in a person living with dementia:

I always had problems with word agitation. I used that word, but (the person living with dementia) was not overly demonstrative. After living with __ for 50 years, I knew something was going on. There would be little tells that __ was upset about something, there were never any bouts of throwing, screaming, or stamping feet. It was all very low-key and difficult to say if there was something going on here. A bad situation would look like absolute refusal to do anything, just a total shutdown.

Caregiver opinions on the use of the BESI tablet app were mostly positive about the process of individualizing the word lists on the tablet for agitation descriptors in the persons living with dementia. The pilot implementation of sending suggestions or recommendations near a possible agitation event was also generally received positively with feedback indicating feelings of usefulness for the future for these suggestions and satisfaction with the technology indications. Although interventions were not deemed timely enough to prevent agitation during the study, lower numbers of agitation events suggests that the *heads up* on the wrist device may have alerted the caregivers before behaviors were evident [8].

### Caregiver Insights on the Burden and Stress of Caring for Persons Living With Dementia and Agitation

In total, 2 caregivers indicated difficulty with the technology, noting it was burdensome to “remember exact time of the agitation” if they had been out. However, the functionality of the system did not require them to document any agitation if they were out of the home. This may speak of their commitment to their efforts and the project. Multiple recommendations to improve the system included agreement with the recommendations—“to be able to add to the choice on the Daily report page”—by 70% (7/10) of caregivers. Additional comments by 4 caregivers included “maybe better words...to describe what [the person living with dementia] did,” referring to the words describing agitation available for quick selection on the tablet.

Caregivers were involved in the development of the technology throughout; however, limited recall in the CANIS interviews may indicate the stress and burden of the caregiving process despite feeling supported by the research team. Few mobile apps are available to support caregivers of persons living with dementia with behavioral difficulties [5]. Developing technologies must be adapted to the needs of caregivers.

Burden was also expressed in dealing with the system with physical manifestations of the stress of caregiving and in the acceptance that the disease is progressive, “That’s part of the burden. You have to deal with the agitation, then recording it.” No help in the cascade of caregiver feelings was described with the example of a caregiver saying, “I don’t know anything to help caregiving tasks.” The open-ended responses to the *no help* example were supported with statements such as:

It’s just like nothing helps. You are just in it with the two of you and you need to determine your own ways of dealing with things.

The burden of caregiving was especially evident in 3 caregivers who agreed that caregiving was too difficult for them to imagine anything being of help. Moreover, 6 caregivers disagreed with the *no help* comment, stating that they felt more positive and useful even with physical manifestations, while in the caregiver role:

The stress level [of caregiving] was such that atopic dermatitis kicked in, I think it is difficult to express there is not hope.

You come to assist, but nothing will fix it.

In seeking additional understanding of the home situation, we asked if anything happened to them during the study. We received only 2 responses, and both addressed physical stressors and family stress. Further inquiry into their situations revealed that many had taken actions, including 2 caregivers choosing placement (although one found it more stressful because of making daily visits), 2 adding services in the home, 3 improving their social involvement, and 3 changing their environment—2 within the home setting and 1 relocated, leaving the caregiver role to another family member. These significant changes indicate proactive decisions and changes within the dyads.

Seeking information about caregiving since they completed the study, we asked, “What intervention and prevention strategies have you found most helpful in recent caregiving?” In response, 60% (6/10) of the caregivers gave strategies including:

Telling people to walk away and pick your battles

I tried to identify a trigger like nothing being in the house to eat

Finally, we were interested in whether any complementary or alternative therapies had been tried for either the caregiver or the person living with dementia. In total, 3 had done so. Massage therapy for the person living with dementia was mentioned twice. Aromatherapy, meditation, and light therapy were administered. These caregivers continued to try new techniques to help improve their caregiving situation. Proactive caregivers demonstrated the ability to take action to improve their situation. Quality of life assessments support the positive behaviors demonstrated by caregivers.

### Caregiver Recommendations for System Changes

The interviews addressed system usability. Ease of use applied to multiple facets of the technology system:

Overall *acceptability* was demonstrated in confidence in handling the technology—“They were not a bother to me. I was actually called to fix things myself.” Acceptability was shown in using the system as a tablet, rather than having “the app on the phone...would not make it easier while out.” In total, 70% (7/10) of the caregivers found it was not intrusive. They had no problems with notifications on the smartwatch or with receiving the automated intervention suggestions. The esthetics of the sensors on the walls in the home were not a problem for most but caregivers noted:

It needs to be considered better...knocking them off the wall due to being curious of what they are or malfunctioning of equipment such as “his watch kept messing up.”

Functionality issues increased burden at times. Difficulty using the system yielded concerns with functionality:

It was a nuisance to run over to the tablet and be putting in information every time I thought something was worthy.

Functionality was also identified with caregivers as an area to focus on:

Maybe better words would be good to describe what he did, since I do not think a lot of them fit him.

Difficulties with the developing technology have caused some frustration. Wall sensors were an esthetic problem for 60% (6/10) of the caregivers, with 1 caregiver noting “...units need to be smaller, less intrusive...” Frustration at the rudimentary look of the mounted sensors and the method of attaching it was reported. For example, 1 caregiver noted “times when the sensors did not work*”* and 2 caregivers found the equipment to be intrusive. Our engineer support team and nurse coordinator were available to address these issues and were received very positively in all surveys. However, frustration persisted in the current situation. Caregivers provided good suggestions for the next steps, even with these frustrations indicating negative ease of use:

We ran in to one of the sensors and it fell off, but when we set it back up we did not know if it was still working. There should be some sort of indicator that says if it is working.

### Future Recommendations

The caregivers offered recommendations during the interviews. In total, 70% (7/10) of the caregivers agreed that they would like to add to the choices on the daily report completed on the tablet, and 1 wanted to be able to give their own insights:

A place to put an explanation of what we thought was causing the agitation.

Practical feedback included [making sensors] less intrusive and industrial looking

Innovative recommendations pointed to the user insights and lessons learned and their feelings of usefulness in assisting in the development of the technology system. All caregivers who provided future recommendations (n=9) agreed that the ability to measure what set off the agitation would be helpful. As noted in the cascade of subjective thoughts (phase 2 in the BESI study), when asked about the future ability to measure over time attitude and activities that set off the agitation of the person living with dementia, all 10 caregivers agreed, noting:

Yes, that would be good (with caregivers recommending) on a scale like day 1 to 30 to track progress, or a way to see the trend of behavior over time, and finally, a trendline that says this behavior is becoming more prevalent. Something that would give me a heads up...like something’s developing here, you need to be watching for it.

Other insights for future development included:

Not just this specific thing is happening, and you need to do something about it. But we see something coming over the horizon, tell me about that, and I know the equipment only worked in the home, but maybe a way to gauge external stressors when we are out. We noticed when she watched the news at night that would stress her out. Maybe a reporting mechanism that says what happened.

## Discussion

### Principal Findings

This study evaluated the participation in developing technology that addresses issues important to caregivers of persons living with dementia. We sought caregiver experiences based on mood, burden, personal reflections, and recommendations. Caregiver feedback highlighted the themes of *usefulness* and *helpfulness,* supported by the subthemes of *agreeability* and *ease of use.* The CANIS study helped to further our understanding of the BESI system. We elicited new information from caregivers, including the potential burden with participation, and obtained feedback about the technology.

New efforts to offer technology focusing on caregivers and older adults find that with in-home monitoring [29] or with older adults aged ≥55 years, searching for mental health resources for others [32], there is frustration or difficulty with the resources available [24]. However, they respond positively to the potential of technology to offer proactive approaches [22]. Even younger adults are frustrated with technical glitches and difficulty navigating an app for mental health support [33].

Most caregivers felt that the technology components were *not a bother*, addressing the ease of use of the BESI system. Ease of use proffers that there is functionality in using the BESI system for the early detection of dementia-related agitation. Ease of use is key to new technology, helping with uptake. Thus, if a technology is helpful but not easy to use, there is more resistance or a lack of acceptance. In this study, the importance of the clarity of words used in describing agitation was identified, with caregivers seeking simpler words and more choices. Usefulness was evident in their acceptability and agreeability and confidence in dealing with new technology.

The functionality of the system reflects that the BESI system can detect agitation events and that it is accurate at detecting early agitation [8]. Functionality also addressed caregiver feelings responsible for entering information in the tablet app even if they were away from the home, especially as this was not a requirement of the study. Caregivers saw the potential in the technology and offered recommendations to improve functionality and ease of use. Timeliness, as an element of functionality, is of utmost importance in the delivery of agitation notices in time to intervene. Notifications occurring in the middle of an agitation episode were not useful. Simplicity in using the system is also important, and issues noted include sensors falling off walls, button presses, or having to scroll for the page needed on the app.

In addition, 30% (3/10) of the caregivers wanted to record more of their impressions—“I thought a place to put an explanation of what we thought was causing the agitation.” This highlighted the theme of helpfulness and could be an added component in future development of the BESI system. Some studies involve caregiver journals and diaries [34,35] for caregivers to record their thoughts. Web-based caregiver forums are beneficial to caregivers [36]. Our focus was on the technology, the acceptance of the technology by the caregiver and person living with dementia, and the potential help it will provide with further development by providing journaling capabilities or other formats of web-based caregiver support within the BESI tablet app.

Future expansion of the process of automating personalized interventions developed with BESI is also needed. Caregivers agreed that the ability to measure what set off the agitation would be helpful. Most caregivers indicated potential, indicating that a proactive mindset would be helpful. Recent work with formal and informal caregivers in the use of unobtrusive monitoring in the home brought forth themes of prevention and proactive measures as helpful [7]. Future potential of the technology could include addressing stress, strain, and the burden of caregiving. Even with the increasing number of technologies available for use for older adults [2], behavioral disturbances or agitation are rarely addressed specifically, but the need for this is supported by this study. Helplessness was identified in a study of mHealth apps related to the vast amount of digitally available information [5]. Strategies were used to choose simplicity of look and ease of use over the level of information available on the apps [5]. The importance of user involvement in the development of technologies is essential in providing appropriate systems that empower users in negotiation of information for health care challenges for themselves or those for whom they care.

The well-received intervention suggestions and decreased number of agitations confirmed that BESI has good value and acceptability. The system’s future capability was supported by noting the potential usefulness of a system in offering timely notifications of an impending agitation episode. The importance of assisting family caregivers with nonpharmacological support for managing behavioral symptoms in dementia has been prioritized for future research [37]. Persons living with dementia in different disease stages with agitation behaviors responded differently to the proposed intervention. The interventions with positive ratings varied between dyads, highlighting the need for personalized dementia-related interventions in CANIS.

The caregivers were able to be present almost all the time. They demonstrated personal investment in the care that the person living with dementia received. Their willingness to participate in, work with researchers, and use technology 24 hours a day for 30-60 days was a significant commitment. Many were not necessarily technologically proficient caregivers, but they wanted to be useful. None of the caregivers implied that the research should not continue to be refined.

Caregiver feedback postdeployment about the BESI system and their overall experience was generally positive, indicating caregiver acceptance of the technology system (in-home sensors, actigraphy or smart watch technology, and tablet app). Caregiver acceptance of developing technology was consistently demonstrated by tolerance, commitment, and their efforts to offer recommendations related to ease of use, functionality, and future capability. Many offered specific suggestions and recommendations, including interest in a journaling format that could inform the next phase of BESI system development.

Finally, assessment of caregiver depression, burden, and caregiver insights into depression and quality of life for the person living with dementia supports the need for caregiver help and support when handling dementia-related agitation. The abovementioned themes support the positive process of involving caregiver knowledge and experience to inform further development of a potentially helpful technology. Using the Progressively Lowered Stress Threshold model as a framework provides a tool to help caregivers better understand agitation triggers and their effects on persons living with dementia as the disease progresses. With more disease burden, smaller triggers will be more important to identify, thus helping to reduce stress in the environment and prevent serious agitation.

### Limitations

Although this study provides several valuable insights, several limitations must also be noted. First, although it was positive to have half of the previous dyads participate in this extended study, the sample size is small and offers limited generalizability. Because interviews occurred a long time after the start of phases 2 and 3 participation (mean 506.47 days, SD 424.52; *R*=49), some noted that they forgot information or were unable to recall details. It may be that opinions and reactions would have been different if all caregivers were interviewed sooner after their deployment experience.

The agitation detection system used to notify caregivers is based on monitoring the behaviors of the person living with dementia using wearable and environmental sensors. Thus, the agitation detection system may miss subtle agitated behaviors, such as when the person living with dementia stays still and refuses care. The detection system also learns the agitated behaviors of persons living with dementia based on the caregivers’ observations and reports. A late report of agitation by the caregiver, which can be caused by the need for immediate attention and intervention required to stop agitation from escalating, may cause delayed detection and notification of the reported agitation. The system can be tuned to notify caregivers of agitation earlier, but this may cause more false alarms.

Finally, although the purpose of delivering interventions in this pilot study was received positively, the process needs refinement and enhanced timeliness. For example, the system only provides an agitation intervention suggestion list to caregivers via a tablet device. Some caregivers may find the checking-on-tablet inconvenient and may prefer that the intervention list be sent to their mobile phone if they have the habit of carrying the phone with them.

### Conclusions

Dementia caregivers dealing with agitation demonstrated acceptance of this developing technology by their initial participation in 30- and 60-day or 60-day deployments and allowing follow-up interviews months afterward. The caregivers consistently demonstrated tolerance, commitment to using the technology, and offered extensive feedback on ways to improve the system. The themes of usefulness and helpfulness were discerned and support the use of caregiver knowledge and experience to inform further development of the technology. Ease of use and acceptability were the subthemes revealed in the analysis. The importance of caregiver involvement in the development and implementation of new systems is essential to provide useful and acceptable technologies. Future development of technologies such as this is especially needed to support caregivers in dealing with behavioral disturbances caused by dementia. These developments could help to reduce the significant stress and burden that caregivers of persons living with dementia live with.

## Appendix

Multimedia Appendix 1 Assessment tool descriptions for creating individualized recommendations for both caregivers and persons living with dementia.

## Abbreviations

BESI: Behavioral and Environmental Sensing and Intervention
CANIS: Caregiver-Personalized Automated Non-Pharmacological Intervention System
CSI: Caregiver Strain Index
