# Supplementary material for: Dementia Caregiver Experiences and Recommendations for Using the Behavioral and Environmental Sensing and Intervention System at Home: Usability and Acceptability Study
Source: JMIR Aging. 2021 Dec 6;4(4):e30353. doi: 10.2196/30353 (PMC8691404; doi:10.2196/30353)
Supplement: Multimedia Appendix 1 [file aging_v4i4e30353_app1.pdf]

CANIS Multimedia Appendix 1: Assessment tool descriptions for creating individualized recommendations for both caregivers and persons living with dementia

MEASURES CAREGIVER STATUS				
**Scale**	**Scale Description**	**Variable**	**Total Score**	**Scoring Level**
CES-D	Depression in caregivers. rarely=0, some=1, occasional=2, most=3; Total out of 60.<br>Higher score= more symptomatology	S<br>Dep	60	Lower = Less depression symptomology
PSQI	7 components are each scored from 0 (no difficulty) to 3 (severe difficulty) then added. The total global score ranges from 0 to 21, with higher scores for worse quality of sleep, >5 indicating poor sleep quality as well as severe difficulties in at least two areas, or moderate difficulties in more than three areas.	S	21	Lower = Better sleep quality
RSSE-respite	0-100% confidence in rating efficacy/ confidence today in 3 categories, obtaining respite.	S<br>Emp	100	Higher = Greater self-efficacy obtaining respite
RSSE-behaviors	0-100% confidence in rating efficacy/ confidence today in 3 categories, responding to disruptive patient behaviors.	S<br>Emp	100	Higher = Greater self-efficacy responding to behaviors
RSSE-thoughts	0-100% confidence in rating efficacy/ confidence today in 3 categories, controlling upsetting thoughts about caregiving.	S<br>Emp	100	Higher = Greater self-efficacy controlling upsetting thoughts
Zarit	Measures caregiver burden: ≥17/48 severe/high burden, 0-12 little or no burden, 13-24 mild to moderate, 25-36 moderate to severe, 37-48 severe	S	48	Lower = Less feelings of caregiver burden
CSI	CG strain index: Any score 7 or higher indicates high level of caregiver strain (in CANIS study only)	S	13	Higher = Greater caregiver strain, ≥7 = strain

MEASURES STATUS IN PERSONS LIVING WITH DEMENTIA				
**Scale**	**Scale Description**	**Variable**	**Total Score**	**Scoring Level**
3MS	A measure of cognitive impairment. Total = 100 points	CI	100	Lower = More impairment<br>< 79 = cognitive impairment
NPI-Q SS	NPI-Q consists of the Symptom Severity score and the Caregiver Distress score to any of 12 symptoms present in the last month. The total NPI-Q severity score (0-3) represents the sum of individual symptom scores and ranges from 0 to 36.	Ag	36	Lower = Less severe symptoms<br><br>Mild = 0-12, Mod = 13-24, severe = 25-36
NPI-Q CGD	NPI-Q consists of the Symptom Severity score and the Caregiver Distress score (0-5). The total NPI-Q distress score represents the sum of individual symptom scores and ranges from 0 to 60.	Beh	60	Lower = Less caregiver distress<br><br>Mild = <25, Mod = 25-36, severe = >36
CMAI-C frequency	2 measures: frequency of behaviors (highest score is 203)	Ag	203	Lower = Less frequent occurrence of behavior in past 2 weeks
CMAI-C behavior occurrence	2 measures: occurrence of behaviors that score ≥2, which means behavior occurred. The total number of behaviors with scores ≥2 is out of 29.	Ag	29	Lower = Fewer behaviors that occur at all
CDS	Rates depression in PWD (by caregiver observation of severity) as well as interview and observation of PWD.<br>0(absent), 1(mild-intermittent), 2(severe).	CI<br>Dep	38	Lower = Less depression,<br>>12 = Probable depression
QoL-AD	Poor=1, Fair=2, Good=3, Excellent=4. Total out of 52<br>Contains 2 measures – one each from the caregiver and the interviewer	Beh	52	Higher = Greater quality of life<br>Poor = <13, Fair = 13-26, Good = 27-39, Excellent= 40-52.
Barthel	Scores: 0-20 = "total" dependency, 21-60 = "severe" dependency, 61-90 = "moderate" dependency, and 91-99 = "slight" dependency. Most studies apply **the 60/61 cutting point**, with the stipulation that the Barthel Index should not be used alone for predicting outcomes. 100 x (observed score-minimum score)/score range	Beh	100	Higher = Greater independence in ADLs
CDR	Clinical Dementia Rating<br>Average of score from each section out of 3	CI	3	Lower = Less impaired
FAST	Stages normal to severe Alzheimer's Disease (1-7). For statistical analysis, each stage is converted to a numerical value (0-16)	CI	16	Higher = More impairment

PSQI	7 components are each scored from 0 (no difficulty) to 3 (severe difficulty) then added. The total global score ranges from 0 to 21, with higher scores for worse quality of sleep, >5 indicating poor sleep quality as well as severe difficulties in at least two areas, or moderate difficulties in more than three areas.	Beh<br>Dep<br>CI	21	Lower scores = Better sleep quality

Variable abbreviations:
Ag – Agitation; Beh – Behavior; CI – Cognitive Impairment; Dep-Depression; Emp-Empowerment; S-Stress/Strain

Assessment scale abbreviations:
CES-D: Center for Epidemiologic Studies Depression Scale; PSQI: Pittsburg Sleep Quality Index; RSSE: Revised Scale for Caregiving Self-Efficacy; Zarit: Zarit Burden Scale-short for; CSI: Caregiver Strain Index; 3MS: Modified Mini-Mental Exam; NPI-Q: Neuropsychiatric Index-Brief (SS-Symptom Severity, CGD-Caregiver distress); CMAI: Cohen-Mansfield Agitation inventory – Community, CDS: Cornell Scale for Depression in Dementia; QoL-AD: Quality of Life-Alzheimer's Disease; CDR: Clinical Dementia Rating Scale; FAST: Functional Assessment Staging in Alzheimer's disease.
